# Low Birth Weight of Newborns: Magnitude of the Problem Seen in a 100 Bed Hospital of a Rural Area in Vellore District, Tamil Nadu (India)

**DOI:** 10.4103/0970-0218.66861

**Published:** 2010-04

**Authors:** Kavitha Balaji, Sathish Sankar, Balaji Nandagopal

**Affiliations:** 1 Department of Obstetrics & Gynecology, Sri Narayani Hospital and Research Centre, Thirumalaikodi, Sripuram, Vellore-632 055, Tamin Nadu, India. E-mail: director@snhrc.org; 2 Division of Biomedical Research, Sri Narayani Hospital and Research Centre, Thirumalaikodi, Sripuram, Vellore-632 055, Tamin Nadu, India. E-mail: director@snhrc.org

Sir,

Low birth weight (LBW) has been defined by the World Health Organization (WHO) as weight at birth of less than 2500 g.(1) LBW at birth may be the outcome of either preterm birth (before 37 weeks of gestation) or retarded fetal (intrauterine) growth.(1) LBW is associated with multiple problems such as fetal and neonatal mortality or morbidity. Compromised growth and cognitive development, with increased risk of cardiovascular and metabolic disorders in adult life, has also been reported.(2) The estimated proportion of LBW infants in India is 7.8%.(1) Accurate and reliable data on LBW incidence in India is still limited especially from rural areas of Tamil Nadu.

We undertook a retrospective analysis of LBW data available at the Sri Narayani Hospital and Research Centre, a 100 bed hospital situated outside Vellore city limits. The women who attended the ante-natal clinic or came for delivery were from the rural and peri-urban population of Vellore district of Tamil Nadu, India. We also investigated the association between LBW, random blood glucose level (RBS), hemoglobin (Hb) levels, maternal age and complications during delivery of the patients. Generally, information from rural areas of India is sparse in published literature.

Information on all pregnant women who came for pre-natal check-ups and who delivered a child at the obstetrics and gynecology facility in the hospital during the period January 2005 to October 2008 were included in the analysis. Relevant information was acquired from the medical charts. Hemoglobin level was estimated by the cyanmethemoglobin method. The weights of the newborns were recorded using an appropriate balance (Phoenix Co, India). All babies were weighed within one hour of birth by a trained nursing personnel. The blood glucose levels were measured by GOD-POD method in a fully automated biochemistry analyzer (Dade Behring, Germany).

Statistical analysis was done by comparison of proportions using the Chi-square test in the Epi Info ver.6.03 programme.

Among 1024 subjects studied, a total of 121 (11.81%) newborn babies were less than 2,500 g. There were 304 mothers from peri-urban areas and 720 mothers from rural areas. Among the mothers who had newborn babies with LBW, 36 (11.84%) belonged to peri-urban population and 85 (11.08%) belonged to rural population. There was no statistically significant difference. The widespread problem is a reflection of the lower socio-economic status of rural and peri-urban women affecting their nutritional status. Similar findings have been reported from some parts of India.(3) An LBW prevalence of 39.1% was found in urban areas of Delhi, the maternal age was identified as a significant determinant.(4) In our study, no difference was found in maternal age distribution of rural or peri-urban communities.

The range of age of mothers who delivered normal weight babies was 17–32, median-20, and for those who delivered LBW babies was 21–30, median-22. No obvious difference in maternal age was found. In our series, we did not find any newborn in the category of extremely low birth weight infants, as defined in the literature.(2) Data on LBW related features are shown in the Table 1. In the present study, among the 924 of 1024 mothers screened for hemoglobin (Hb) level, it was more than 10.9 mg% in 35.28% only. Thus, two-thirds of the mothers in this study were anemic. Anemia in the mother is a risk factor for LBW.(5) The random blood sugar level among rural people ranged from 2.7 to 10.6 mmol/l with a median of 5.13 and among the urban people it ranged from 2.7 to 12.7 mmol/l and median was 5.05. Four women alone were identified as potentially having gestational diabetes but no further investigations were done. Among rural women 264 mothers had LSCS, seven mothers had intra-uterine death (IUD), there were two pre-term deliveries, nine of them delivered babies weighing more than 4.0 kgs and 85 mothers delivered babies weighing less than 2.5 kgs. Whereas, in urban mothers, 131 had LSCS, two had IUD and no preterm births, 36 mothers delivered babies less than 2.5 kgs. This indicates the poor nutritional status in rural and urban areas of India as a risk factor for the low birth weight incidence.

**Table 1:** Characteristics of mother and LBW babies stratified as rural *vs*. peri-urban origin

Characteristics	Rural	Urban
Mothers (n)	720	304
Maternal age (years)		
Range	17–35	16–34
Median	23	23
Hemoglobin (mg%)		
Range	4.9–14.9	4.9–14.2
Median	10.6	10.5
RBS (mmol/l)		
Range	2.7–10.6	2.7–12.7
Median	5.13	5.05
Mode of delivery		
Normal	419	176
Normal with episiotomy	22	0
Breech	0	1
Forceps	5	6
LSCS	264	121
Baby weight (grams)*		
1000–2499	85	36
>2500	627	266

* Weight at birth is not known for one child born of a rural mother

Birthweight remains an important factor affecting infant andchild mortality. Babies of low birth weight are also prone to perinatal morbidity. The results of this study suggest that for reducing the problem of LBW in rural India, the public health strategy needs to focus attention on better maternal nutrition and education. Indian intervention programs encourage wider birth interval, avoidance of teenage marriages and early pregnancies. The public health measures have been most effective only in the state of Kerala; other parts of the country have to play catch-up.
